# Systems biology-guided identification of synthetic lethal gene pairs and its potential use to discover antibiotic combinations

**DOI:** 10.1038/srep16025

**Published:** 2015-11-04

**Authors:** Ramy K. Aziz, Jonathan M. Monk, Robert M. Lewis, Suh In Loh, Arti Mishra, Amrita Abhay Nagle, Chitkala Satyanarayana, Saravanakumar Dhakshinamoorthy, Michele Luche, Douglas B. Kitchen, Kathleen A. Andrews, Nicole L. Fong, Howard J. Li, Bernhard O. Palsson, Pep Charusanti

**Affiliations:** 1 Department of Microbiology and Immunology, Faculty of Pharmacy, Cairo University, Cairo, 11562, Egypt; 2 Department of Bioengineering, University of California, San Diego, La Jolla, California, 92093, USA; 3 Computer-Aided Drug Discovery, Albany Molecular Research, Inc., Albany, New York, 12203, USA; 4 Albany Molecular Research Singapore Research Centre, Pte Ltd, Singapore, 117525; 5 The Novo Nordisk Foundation Center for Biosustainability, Technical University of Denmark, 2970 Hørsholm, Denmark

## Abstract

Mathematical models of metabolism from bacterial systems biology have proven their utility across multiple fields, for example metabolic engineering, growth phenotype simulation, and biological discovery. The usefulness of the models stems from their ability to compute a link between genotype and phenotype, but their ability to accurately simulate gene-gene interactions has not been investigated extensively. Here we assess how accurately a metabolic model for *Escherichia coli* computes one particular type of gene-gene interaction, synthetic lethality, and find that the accuracy rate is between 25% and 43%. The most common failure modes were incorrect computation of single gene essentiality and biological information that was missing from the model. Moreover, we performed virtual and biological screening against several synthetic lethal pairs to explore whether two-compound formulations could be found that inhibit the growth of Gram-negative bacteria. One set of molecules was identified that, depending on the concentrations, inhibits *E. coli* and *S. enterica* serovar Typhimurium in an additive or antagonistic manner. These findings pinpoint specific ways in which to improve the predictive ability of metabolic models, and highlight one potential application of systems biology to drug discovery and translational medicine.

Genome-scale metabolic network reconstructions form a cornerstone of microbial systems biology as they capture in one framework all known genes, proteins, and reactions within the metabolic network of an organism^1^,^2^. The subsequent conversion of a reconstruction into a mathematical model suitable for numerical computation allows one to compute cellular phenotypes for different growth conditions. In this way, metabolic reconstructions and their associated models constitute a computational platform that can be used to investigate the link between genotype and phenotype. This property distinguishes a metabolic model from static maps of biochemical pathways. The latter is a catalog of all known pathways in a network, whereas the former provides additional information such as growth rates in different environments; substrate uptake and product secretion rates; and the identity of pathways that are likely to be active for a given growth condition versus pathways that are present but not utilized. Such models have found use in metabolic engineering^3^,^4^, network analysis^5^,^6^, and have driven biological discovery^7^,^8^.

For well-characterized organisms such as *Escherichia coli*, *Bacillus subtilis*, and *Saccharomyces cerevisiae*, metabolic models simulate single gene essentiality with overall accuracy of approximately 90%^9^,^10^,^11^,^12^. This rate drops to 49%, however, when the *S. cerevisiae* iLL672 metabolic model is used to compute synthetic lethality instead^13^. Although this rate is two orders of magnitude better than if two genes are selected by random chance^13^, it still represents a drop-off when compared to the ~90% accuracy rate for single gene essentiality. The simulation accuracy of synthetically lethal (SL) gene pairs for the *E. coli* metabolic model^10^ is not known since it is much more difficult to create large numbers of double deletion mutants in *E. coli* than it is in *S. cerevisiae*. Correct simulation of synthetic lethality, and more generally gene-gene interactions, therefore constitutes one area in which metabolic models can be greatly improved.

With greater biological accuracy, the *in silico* computation of synthetic lethal gene pairs could become one strategy to rationally discover combination therapeutics. Currently, some common strategies to discover drug combinations include: the inhibition of multiple key steps in pathways known to be essential, such as folate biosynthesis^14^,^15^; the inhibition of an essential enzyme and another protein known to confer resistance, as is the case with Augmentin^16^; and high-throughput screening of a compound library in the presence of a second compound^17^,^18^. The inhibition of synthetic lethal proteins has been applied to the field of cancer therapeutics^19^,^20^, most notably the inhibition of poly(adenosine diphosphate [ADP]–ribose) polymerases (PARPs) in different cancers^21^,^22^,^23^. Specifically, PARPs and *BRCA1*/*BRCA2* form a synthetic lethal pair in cancer cells, but in some cases the latter has developed a loss-of-function mutation such that only one compound targeting the PARPs needed to be developed. The situation would differ for antibiotics: two compounds targeting both members of the SL pair would likely be needed. This strategy becomes more attractive if the two molecules act synergistically, since synergy can result in lower individual dosages of the two compounds and possibly more target specificity^24^. Metabolic models can significantly accelerate the search for SL gene pairs. To take *Escherichia coli* K12 MG1655 as an example, a strategy based on the inhibition of two different enzymes would yield a total search space of approximately 8 million protein pairs since this strain has 4140 annotated coding DNA sequences (CDSs). In practice, however, the search for potential targets within this space is more focused, and metabolic models can narrow the search space even further due to their ability to simulate the effect of gene knockouts on growth.

We analyze here how accurately the most current version of the *E. coli* metabolic model^10^ computes synthetic lethality, and identify the most common failure modes to pinpoint specific areas in which the model can be improved. Furthermore, we perform virtual screening against a subset of validated SL pairs as a first step to find potential inhibitors, and then screen the most promising compounds both as single agents and in combination to assess the feasibility with which combinations of inhibitors can be found against the SL proteins. In the combination studies, we also assess whether the inhibitors exhibit synergy, additivity, or antagonism, and examine whether these effects match the expected outcome based on their protein targets and simulation data.

## Results

### Metabolic network models can guide the identification of synthetic lethal gene pairs

**Figure 1:**
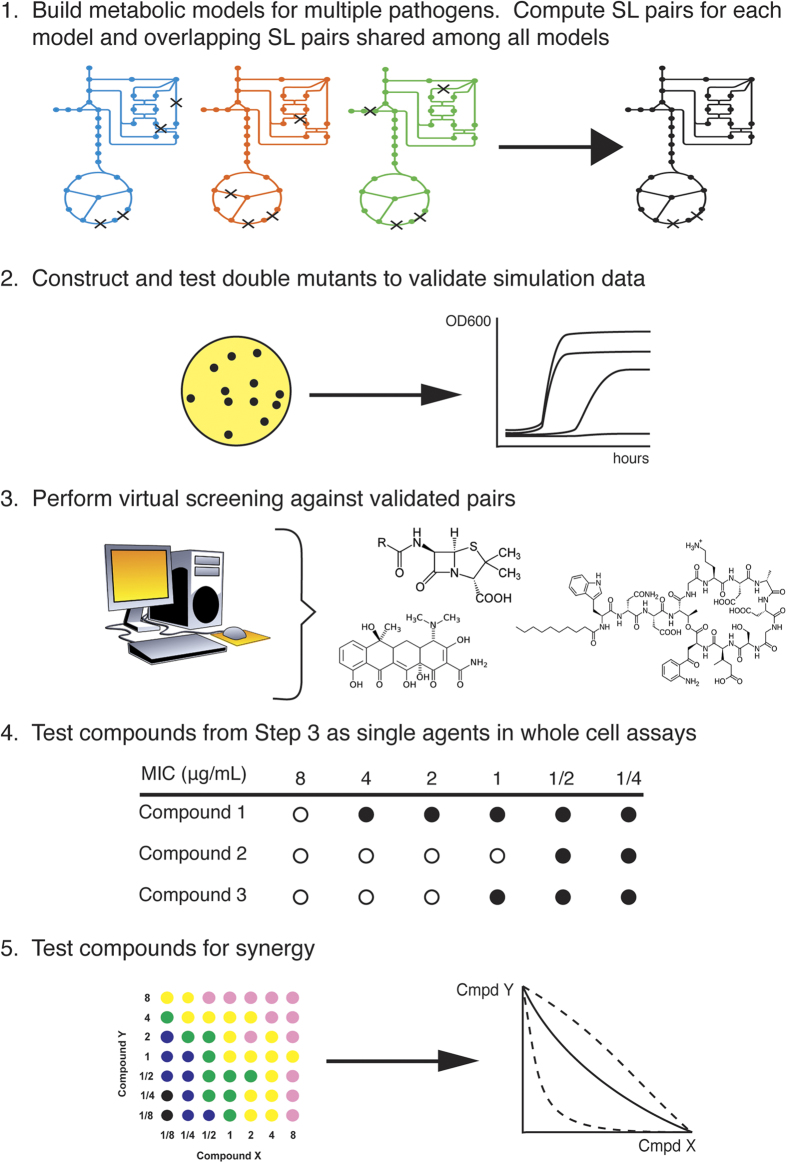
Five-step workflow for the model-guided identification of compounds that inhibit synthetic lethal protein pairs. The use of mathematical models in step 1 narrows the search space for SL pairs, after which the pairs are validated experimentally and then subjected to virtual and biological screening.

We used metabolic network models for *E. coli* EDL933^25^, *K. pneumoniae* MGH78578^26^, *Y. pestis* CO92^27^, and *Salmonella enterica* serovar Typhimurium LT2^28^ (hereafter referred to as *S.* Typhimurium) to guide our search for possible synthetic lethal pairs in these organisms. Other model-driven studies of synthetic lethality in *E. coli* have used metabolic models of the non-pathogenic K12 MG1655 strain^29^,^30^; however, we used a model for strain EDL933 to allow for potential application to infectious disease and drug discovery (Fig. 1). This strain is a prototypical one for the enterohemorrhagic O157:H7 serotype that has caused multiple outbreaks of food poisoning in the United States^31^. The strains chosen to represent the three other bacteria are likewise pathogenic variants for those organisms.

**Figure 2:**
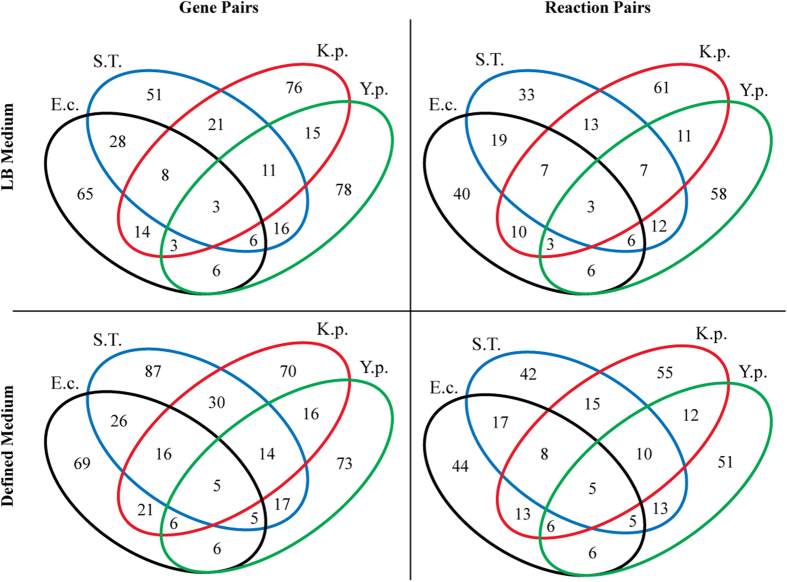
The number of SL genes and their corresponding reactions in each of the four individual models, and the number that were shared among multiple models. BCS media was the defined medium for the *Y. pestis* model whereas glucose M9 was the defined medium for the other three. Abbreviations: E.c.: *E. coli* EDL933; K.p.: *K. pneumoniae* MGH78578; Y.p.:*Y. pestis* CO92; S.T.: *S.* Typhimurium.

In LB media, simulations of the four models resulted in 65 (*E. coli*), 76 (*K. pneumoniae*), 78 (*Y. pestis*), and 51 (*S.* Typhimurium) computed SL pairs. Of these, three pairs are shared among all four models: *asnA*/*asnB* (amino acid metabolism), *cmk*/*pyrH* (nucleotide salvage pathway), and *tdk*/*thyA* (nucleotide salvage pathway). After these three pairs are removed, eight, five, three, and zero pairs are shared among three of the four models, respectively. These pairs fall predominantly into the following classifications: folate metabolism, amino acid metabolism (asparagine, methionine, and aromatic amino acids via the shikimate pathway), cofactor biosynthesis (pantothenate), and TCA cycle. These 19 SL gene pairs correspond to 13 unique reaction pairs, while there were six cases in which two or more SL gene pairs catalyzed the same reaction in the models. For example, *lpdA*/*sucC* and *lpdA*/*sucD* are two distinct SL gene pairs, but they correspond to only one SL reaction pair since SucC and SucD form a heterodimer. These data are summarized in Fig. 2.

In defined media, the number of computed SL pairs was 69 (*E. coli*), 70 (*K. pneumoniae*), 73 (*Y. pestis*), and 87 (*S.* Typhimurium). The defined medium for *Y. pestis* was BCS media^32^, while the recipe for glucose M9 was used as the defined medium for the other three organisms. Five pairs were shared among all four models, *asnA*/*asnB* and *cmk*/*pyrH* as in LB medium plus *glyA*/*serA*, *glyA*/*serB*, and *purN*/*purT*. Unlike in LB, simulations did not identify *tdk*/*thyA* as a putative SL pair in defined media because *thyA* is singly essential under these conditions. Besides these five pairs, 21 other SL gene pairs were present in at least three of the four models. They again belong predominantly to folate metabolism, amino acid metabolism, and TCA cycle, with several additional pairs from ion transport and the pentose phosphate pathway. Overall, the 26 SL gene pairs correspond to 16 unique SL reaction pairs. These data are likewise summarized in Fig. 2. The full list of SL gene pairs for both LB and defined media can be found in Supplementary Dataset 1.

### The overall accuracy of model-guided prediction of synthetic lethality was between 25% and 43%

We tested how accurately the models could predict synthetic lethality *a priori* by constructing eight double knockout mutants in *E. coli* EDL933 and four in *Salmonella enterica* strain 14028s. In *E. coli*, one of the eight double mutants was experimentally synthetically lethal (*hemF*/*hemN*), whereas in *Salmonella* it was two out of four (*gltB*/*gdhA* and *lpdA*/*sucC*) (Table 1). Therefore, the overall accuracy was three out of twelve (25%). Five of the false positives resulted from cases in which one of the two genes was singly essential, and therefore synthetic lethality is not possible. If these are removed from the calculation, the accuracy rate is three out of seven (43%). No proper benchmark of these results exists since a systematic, large-scale study that examines the number of SL pairs in *E. coli* has not been carried out yet; however, of 692,865 randomly chosen gene pairs in yeast that were experimentally tested, only 0.56% displayed a synthetically lethal or sick phenotype^13^. If a similar ratio holds for *E. coli*, then the models will have outperformed random selection by a statistically significant amount.

Different mechanisms lead to the lethal phenotype in the three identified SL pairs. HemF and HemN both catalyze the same essential oxidative decarboxylation step during porphyrin biosynthesis, but HemF is active under aerobic conditions while HemN is active under anaerobic conditions. Their removal consequently eliminates an essential reaction. GltB and GdhA catalyze parallel reactions within glutamate biosynthesis: the deletion of *gltB* and *gdhA* removes all possible routes from 2-oxoglutarate to L-glutamate, leading to glutamate auxotrophy. LpdA is a component of two separate complexes that catalyze different steps within the TCA cycle, and SucC is the β-subunit of succinyl-CoA synthetase, also within the TCA cycle. The deletion of these two genes severely reduces flux through this critical pathway, thereby accounting for the SL phenotype.

### Incorrect computation of single gene essentiality and missing biological information were the two most common causes of inaccurate SL predictions

We analyzed the nine cases in which the predicted SL phenotype proved to be inaccurate to determine the underlying cause, since such information reveals areas for further improvement of the models. The two most common causes were situations in which one of the two genes in the pair turned out to be singly essential, and missing biological information that consequently was not represented in the models.

There were five cases in which at least one of the two genes is singly essential (Table 1), but the reasons for the discrepancies with simulation data varied. In one case, the two genes (*aroE*/*ydiB*) catalyze the same essential reaction in the model, but *aroE* is singly essential in LB. RNA-seq data indicate that *ydiB* is not expressed during exponential growth in LB^33^,^34^, implying that missing regulatory information that is beyond the scope of metabolic models is responsible for this error. Likewise, missing regulatory information centered on the glyoxylate shunt underlies the error with *mdh*/*ppc*. In contrast, in two cases (*metL*/*thrA* and *fabA*/*tesB*) the non-essential genes (*thrA* and *tesB*) are expressed under the growth conditions expected to result in synthetic lethality. This scenario points to incorrect assignment of gene function as a likely cause. In the last case (*glyA*/*serA*), both genes were singly essential.

There were three cases in which missing biological information led to erroneous simulation results. Simulation of a *purT*/*purN* double knockout resulted in a model for *E. coli* that could not synthesize purine, an essential metabolite; however, the double knockout was viable in LB medium (but not glucose M9), an experimental outcome that has been seen previously in other strains of *E. coli*^35^. These data suggest the presence of an unidentified purine transporter. In the model, the two proteins MutT and NtpA catalyze the same essential reaction within folate biosynthesis, forming the basis for their computed synthetic lethality. The experimental viability of this double mutant strongly suggests that at least one other unidentified protein can catalyze the same reaction. Similarly, ArgD and CstC are putative paralogs that also catalyze the same essential reaction in the model.

### Validated synthetic lethal pairs can guide the search for possible combination therapeutics

We performed virtual and biological screening to identify potential inhibitors against four sets of proteins: *hemF*/*hemN*, *lpdA*/*sucC*, *glyA*/*serA*, and *mdh*/*ppc*. The first two are synthetic lethal pairs (Table 1), and compounds that inhibit them were hypothesized to act synergistically. The latter two were screened for use as controls. The *glyA*/*serA* pair is a case in which both genes are singly essential in *S.* Typhimurium, so they were anticipated to exhibit additivity instead of synergy. The *mdh*/*ppc* pair is a case in which one gene (*ppc*) is singly essential. Consequently, neither synergy nor additivity was anticipated for this pair. We followed the following general strategy to identify potential combinations of inhibitors: 1) perform virtual screening against the enzyme targets; 2) screen the compounds against *E. coli* and *S.* Typhimurium to determine their activity as single agents; and 3) screen a subset of the compounds in combination according to the SL pairing to evaluate potential synergy, additivity, and antagonism. It should be noted that, for true SL pairs, compounds that inhibit either of the two proteins are not expected to show strong activity as single agents. Instead, inhibition is expected to be strongest when both compounds are present.

We first used molecular docking to identify compounds likely to inhibit the eight proteins in either *E. coli* or *S.* Typhimurium as single agents. A diverse collection of 300,000 to 600,000 compounds was docked to binding sites within each of the eight proteins using a high throughput docking procedure (HTVS mode, see Supplementary File 1). High scoring compounds from these searches were used as similarity probes against a 70 million compound library and the hits were docked in a higher precision mode (SP mode). This step resulted in a set of 1,039 compounds.

The 1,039 compounds were purchased and tested in a 10-point dose response format (top test concentration of 200 μM and 2-fold dilution) to determine their IC_50_ and % inhibition against the two bacteria. Although virtual screening was carried out against protein targets, this step was performed as a bacterial growth inhibition assay in which the readout was cellular growth. Standard compounds were also tested along with every batch of compounds screened in the assay for quality control purposes. This step identified 67 compounds that exhibited over 20% inhibition at any dose.

These 67 weak hits were then used as probes to search for chemically similar structures that might also be active, and these compounds were likewise docked against the eight target proteins. This step resulted in a group of 289 compounds that were also purchased and screened in the cellular assays. Therefore, a total of 1328 compounds were identified by virtual screening and tested in the first round of biological screening as potential inhibitors against *E. coli* and *S.* Typhimurium (Table 2). After a hit was identified against one protein (% inhibition > 20% against either bacterium), it was subsequently docked against all eight proteins to assess whether it might bind to the other targets as well. If the docking score was at least one standard deviation above the average score for that site, then it was presumed to bind that additional site. A third qualitative assignment was made if the scores from two methods were deemed significant (see Materials and Methods). These multiple assignments are listed in Table 3 for the most active compounds.

Three of the 1328 compounds showed reproducible activity against *E. coli*, *S.* Typhimurium, or both during individual compound screening with IC_50_ < 200 μM and/or percent inhibition over 30% (Table 3), and we selected them for combination studies. SERA-126 inhibited the growth of both bacteria. Its IC_50_ value was lower for *S.* Typhimurium than it was for *E. coli*. SIM1-074 inhibited the growth of *S.* Typhimurium but did not have significant effect on the growth of *E. coli*. SIM4-003 inhibited the growth of both *E. coli* and *S.* Typhimurium with IC_50_ values of <50 μM.

From the docking calculations against all eight proteins, combinations of these three compounds were presumed to target three of the four protein pairs (Table 3); however, the calculations suggested that SerA/GlyA would be the primary target. SERA-126 and SIM4-003 most likely target SerA and SIM1-074 is predicted to inhibit GlyA. This pair was not an experimentally valid SL pair because the two genes are singly essential (Table 1). Although SIM1-074 targets SucC in the calculations, combinations of these three compounds were not predicted to inhibit SucC/LpdA because SERA-126 and SIM4-003 are predicted to inhibit LpdA only weakly. The compounds exhibited weak inhibition for the HemN/HemF pair in the calculations, and it is equally likely that each compound would bind to HemN only. None of the compounds bound to Ppc in the calculations; therefore, the compounds were not predicted to have an effect on the Mdh/Ppc pair.

Two concentrations of SIM1-074 were tested against two concentrations each of SERA-126 and SIM4-003 against both *E. coli* and *S.* Typhimurium. The chemical similarity between SERA-126 and SIM4-003 suggested that these compounds would share a similar mechanism of action, and therefore they were not tested in combination with each other. The tested combinations were assigned to have either a synergistic, additive, or antagonistic effect in the combination testing using the Loewe model^36^. Because the SerA/GlyA pair was the most likely target, we anticipated additivity as the most likely outcome.

**Figure 3:**
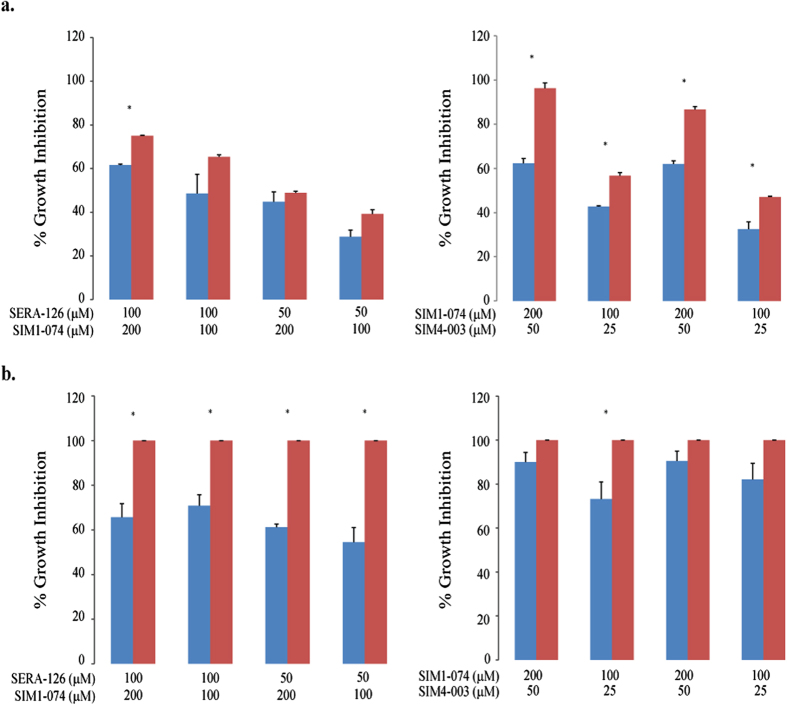
Percent growth inhibition in combination studies against (A) *E. coli* and (B) *S.* Typhimurium. The actual percent growth inhibitions achieved by testing two compounds in combination (blue bars) were compared with expected values based on simple additivity according to the Loewe model (red bars). The standard deviation of the single agent and combination studies ranged as high as 10% but was typically 2–5%. The calculated expected percent growth inhibition for *S.* Typhimurium was greater than 100%, but is represented here as 100%. Asterisks (*) denote statistically significant differences (*p* < 0.05) between the actual and the expected percent growth inhibition using *t*-test.

All combinations exhibited a percent inhibition that was either similar to or lower than the calculated additive values in the combination studies against both strains (Fig. 3). Of note is that the SERA-126/SIM1-074 combination against *S.* Typhimurium resulted in lower than expected percent inhibition by 30–45% (Fig. 3). This effect was outside the experimental uncertainty (3–7%) of the difference of the measurements from the simple additive assumption, and suggests potential antagonism in this case. No synergy was observed.

## Discussion

We present data here that evaluated how accurately systems biology-based metabolic models can predict SL gene pairs in four species of Enterobacteriaceae, especially *E. coli* and *S.* Typhimurium; a workflow for the discovery of combination inhibitors directed against SL pairs; and a set of compound pairs that inhibit growth of *E. coli* and *S.* Typhimurium in plate-based whole cell anti-bacterial assays in an additive manner, which was consistent with the predicted outcome for the presumed SL pair targeted by the compounds. These results highlight specific areas in which metabolic models can be improved and evaluate the ability of the models to guide the selection of anti-bacterial drug combinations.

The overall accuracy rate in identifying SL pairs in this study was 25% to 43%, but the true value is likely different due to several factors. One such factor was the manner in which putative SL pairs were selected for experimental testing. The selections balanced two different goals: to be amenable to the discovery of new inhibitors and simultaneously to provide data about the ability of the models to predict synthetic lethality in an *a priori* manner. Based on these two criteria, the pairs were filtered as follows. First, pairs in which one of the two genes was known to be singly essential experimentally were removed from the list. These pairs are clear incorrect model predictions. Second, pairs in which one member was a membrane-bound protein were removed because such proteins are more difficult drug targets than their cytoplasmic counterparts. There were ten such pairs for the *E. coli* EDL933 model after simulation in M9 medium. Third, pairs already known to be synthetically lethal were removed so that this study remained focused on the discovery of new SL pairs rather than revisit known ones. Nine pairs fit this category for the *E. coli* EDL933 model after simulation in M9 medium, and we ruled out seven of them. We constructed one pair, *purT*/*purN*, because the reported double mutant had additional genetic modification besides the two deletions that might have impacted synthetic lethality. We constructed a second pair, *metE*/*metH*, to explore the limitations of the metabolic models (see below). In the end, we selected from a list of 52 remaining putative SL pairs in *E. coli* EDL933 for experimental testing, and the accuracy rate calculated here was based on this subset. A similar analysis was performed for the *S.* Typhimurium model.

Several specific cases highlight the limitations of metabolic models. For example, a cofactor not accounted for in the models plays an essential role in the reaction catalyzed by MetE/MetH. This pair was predicted by the *E. coli* EDL933 model, as well as the iJO1366 model for the K12 MG1655 strain^10^, to be synthetically lethal in both LB and glucose M9. We tested this prediction by constructing the two single mutants and the double mutant, finding that all three were viable in LB. In glucose M9, however, only Δ*metH* was viable; Δ*metE* and the double mutant Δ*metE*Δ*metH* were both non-viable. Therefore, the double mutant was not a true SL pair since *metE* is singly essential under this condition. Both enzymes catalyze the same essential reaction, but MetH absolutely requires cobalamin to function^37^. MetH is inactive in glucose M9 because cobalamin is not a component of the medium, resulting in the single essentiality phenotype of *metE* under this condition. The metabolic models used here do not explicitly account for details such as cofactors, a deficiency that led to the erroneous prediction of synthetic lethality for this pair. The latest iterations of metabolic models, however, do account for additional features such as gene expression, protein synthesis, and cofactor usage, and therefore correctly predict the requirement for cobalamin for this pair^38^.

Several reasons might account for the low number of compounds identified for each SL protein derived from virtual screening, as only one pair of compounds targeting one SL pair was found. First, the docking methodology has a significant error rate even for well-explored systems, although virtual screening typically identifies compounds at a higher rate than random screening^39^. Second, there were two possible sites of competitive inhibition for many of the enzymes, a cofactor binding site and a second one for the substrate. In these cases, we docked to each site in order to identify a greater number of possible inhibitors. The modifications to the binding sites were intended to mimic reactants, products or transition states in order to fashion the binding site shape appropriately. Third, there were no crystal structures containing an inhibitor bound to the protein for any of the eight proteins, which increases the difficulty of the chemical search.

In a similar fashion, there are several reasons for the lack of activity observed in the growth inhibition assays during testing of both individual compounds and combination testing. The hit rate after testing of individual compounds was ~1%. Factors such as low compound permeability, efflux, off-target binding, and poor target engagement inside the bacterial cell likely contributed to the steep drop-off in the number of compounds that passed from the virtual to the experimental screening steps as these factors are not fully taken into account during virtual screening. Moreover, the screen was performed against wild-type cells. In this design, compounds should not display strong activity when present individually since they would inhibit only one of the two proteins in the SL pair, making it difficult to identify promising agents that might be active in combination. Future studies might approach this problem by screening against knockout mutants rather than the wild-type variant. For example, if two proteins X and Y form an SL pair, one would perform virtual screening against protein X to identify potential inhibitors, and then perform biological screening of those inhibitors using mutants containing deletions in gene Y. One would do the opposite case as well. Active compounds identified in these two screens would have a greater chance of having the expected mechanism of action, inhibition of the target protein in the SL pair, rather than inhibition arising from a different or non-specific mechanism. The compounds would then be subjected to combination studies.

Looking forward, the findings presented here pinpoint specific ways in which metabolic models can be improved, and thereby provide a guide for future experimental work. In turn, models that have greater biological accuracy would increase their utility across many different fields, and would serve as the foundation of a workflow that aims to discover combination inhibitors against SL protein pairs. This task would be difficult to implement without the use of metabolic models as a guide due to the extremely large number of possible protein pairs. Consequently, the discovery of combination therapeutics might become much more feasible as the models are improved and refined screening strategies are adopted.

## Materials and Methods

### Strains and media

All knockout mutants were created in *Escherichia coli* EDL933 (ATCC 700927) or *Salmonella enterica* serovar Typhimurium 14028s (ATCC 14028). Both strains are enteric pathogens. Compound screening was performed against *E. coli* Seattle 1946 (ATCC 25922), which is a Clinical and Laboratory Standards Institute (CLSI) control strain for antimicrobial susceptibility testing, and against *S.* Typhimurium 14028s.

All strains were grown in either Luria-Bertani (LB) broth/agar or glucose M9 media. The M9 medium contained either 2 g/L glucose, 100 μM CaCl_2_, 2 mM MgSO_**4**_, 6.8 g/L Na_2_HPO_4,_ 3 g/L KH_2_PO_4_, 0.5 g/L NaCl, 1 g/L NH_4_Cl, and 250 μL/L trace elements. The trace element solution consisted of (per liter): FeCl_3_•6H_2_O (16.67 g), ZnSO_4_•7H_2_O (0.18 g), CuCl_2_•2H_2_O (0.12 g), MnSO_4_•H_2_O (0.12 g), CoCl_2_•6H_2_O (0.18 g) and Na_2_EDTA•2H_2_O (22.25 g). Antibiotics were added as necessary at the following concentrations: ampicillin at 100 μg/mL, kanamycin at 50 μg/mL, and chloramphenicol at 25 μg/mL. LB powder was purchased from EMD Chemicals (Gibbstown, NJ) and used at the manufacturer’s recommended concentration. All other chemicals were purchased from Fisher Scientific (Waltham, MA) or Sigma-Aldrich (St. Louis, MO).

### Mathematical modeling and prediction of synthetic lethal gene pairs

Metabolic network reconstructions for *E. coli* K12 MG1655 and EDL933^10^,^25^, *K. pneumoniae* MGH 78578^26^*, Y. pestis* CO92^27^, and *S.* Typhimurium LT2^28^ were loaded into and simulated using the python version of the COBRA Toolbox^40^. These models can be downloaded from the BiGG database (bigg.ucsd.edu)^41^. All model reactions retained their default bounds^10^. Both glucose M9 and BCS defined media were simulated by setting a lower bound of each exchange reaction as listed in Supplementary Dataset 1. LB media was likewise simulated by setting bounds on each exchange reaction as indicated in Supplementary Dataset 1.

Each single knockout strain was modeled by using the delete_model_gene function to constrain each reaction catalyzed by the corresponding enzyme to zero. Analogously, double gene deletions were calculated using the double_gene_deletetion_fba module and double reaction deletions were performed using the double_reaction_deletion module. Model growth phenotypes were determined using flux balance analysis (FBA) with the core biomass reaction as the objective. If a particular knockout resulted in a simulated growth rate equal to zero, that gene or set of genes was deemed to be singly essential or synthetically lethal, respectively. As an example, an IPython notebook with command line codes used to carry out this analysis for the *E. coli* K-12 MG1655 iJO1366 model is available at http://nbviewer.ipython.org/github/JonM4024/synthetic_lethals/blob/master/double_deletions_example.ipynb. The Gurobi (Gurobi Optimizer Version 5.6, Gurobi Optimization, Inc.) linear programming solver was used to perform FBA.

### Construction of gene deletion mutants in *E. coli* and *S*. Typhimurium

All gene knockouts were created using the protocol of Datsenko and Wanner^42^. Briefly, a kanamycin resistance cassette containing flanking FRT sites was generated by PCR using pKD13 as the template. The ends of the cassette comprised 60 nucleotides that contained the start or stop codon plus 57 bp that were homologous to the 57 bp immediately upstream and downstream of gene to be deleted. Correct insertion of the marker and subsequent removal from the chromosome were confirmed by PCR and Sanger sequencing. All PCR products were purified with the QIAGEN PCR clean-up kit (Valencia, CA).

### Virtual and Compound Screening

Crystal structures of six proteins were selected from the protein databank (www.rcsb.org) which were identical in sequence to the proteins found in the two bacteria. Two homology models were developed (HemF and LpdA) because no crystal structures were available. In total, thirteen sites were used for docking. Preference was given to those structures that contained a co-crystalized substrate or inhibitor so that we could select compounds that likely bind at a catalytic site. Standardized methods were used to prepare each binding site for docking, and to model the sites with compounds that confer appropriate shape and electrostatic interactions for potential inhibitory compounds. Diverse compounds from a library of 300,000 to 600,000 commercial compounds were docked using high throughput virtual screening (HTVS) precision. The best scoring compounds were subjected to atom pair similarity^43^ calculations to determine chemically similar structures with a similarity cutoff of 70–80%. In addition, compounds that contained the maximum HierS scaffolds^44^ of any compound on the HTVS list were also selected. The resulting combined list of compounds was docked again using standard precision. Compounds that docked well according to the Glide docking program and scored well based on a complementary scoring scheme were examined manually and purchased, if available.

Our selection choices were guided by the need for at least one or two single-agent inhibitors per protein target. Therefore, we set a goal of testing a minimum of 200 compounds per protein target based on reported success rates of selecting true active compounds in typical *in vitro* biochemical screens by docking methods. Docking methods provide a mean enrichment factor of 1–60 over random selections with an enrichment of 10 being a reasonable expectation, or approximately a 10% hit-rate^45^. We also assumed that only ~10% of compounds would permeate the Gram-negative cell walls yielding a net 1% hit rate in bacterial assays.

After identifying weak hits in the bacterial growth inhibition assay, we selected chemically similar compounds and likewise docked them to their putative binding site. The selection of similar compounds was done using atom pair similarity^43^ calculations with a similarity cutoff of 70–80%. These similar compounds were then docked using the same methods as the original compounds. Any resulting compounds that met the same docking score criteria as the original compounds were purchased, if available.

The IC_50_ and percent inhibition for each purchased compound was tested in bacterial growth inhibition assays. All active compounds were re-confirmed by carefully reweighing them to measure percent inhibition and IC_50_ more precisely. Based on the percent growth inhibition data, the best concentrations for combination testing were chosen. A 2×2 matrix of concentrations was chosen such that the simple additive values would be in the 25–75% inhibition range. This choice allows synergy to be distinguished from the combined experimental uncertainty of each compound tested alone.

Tests were also conducted to assess compound turbidity in each of the studies to determine the interference (if any) from the test compounds that could affect the absorbance readings of the assay at 600 nm. None of the compounds had any interference with absorbance at 600nm.

Additional details describing the virtual and compound screening procedures can be found in Supplementary File 1.

## Additional Information

**How to cite this article**: Aziz, R. K. *et al.* Systems biology-guided identification of synthetic lethal gene pairs and its potential use to discover antibiotic combinations. *Sci. Rep.* **5**, 16025; doi: 10.1038/srep16025 (2015).

## Supplementary Material

Supplementary Dataset 1

Supplementary Information 1

## Figures and Tables

**Table 1:** Putative SL gene pairs that were experimentally constructed.

Model-predicted SL pair	Organism	Growth medium on which synthetic lethality was predicted to occur	Experimentally valid SL pair?	Reason for incorrect prediction
*hemF*/*hemN*	*E. coli*	LB	Yes	
*gltB*/*gdhA*	*S.* Typhimurium	M9	Yes	
*lpdA*/*sucC*	*S.* Typhimurium	M9	Yes	
*metE*/*metH*	*E. coli*	LB	No	1
*ntpA*/*mutT*	*E. coli*	M9	No	4
fabA*/*tesB*	*E. coli*	LB	No	3
*purT*/*purN*	*E. coli*	LB	No	4
*ydiB*/aroE*	*E. coli*	LB	No	3
metL*/*thrA*	*E. coli*	M9	No	3
*cstC*/*argD*	*E. coli*	M9	No	4
glyA*/serA*	*S.* Typhimurium	M9	No	3
*mdh*/ppc*	*S.* Typhimurium	M9	No	2,3
*ntpA*/*hemF*	EDL933	Negative control; double mutant was viable on LB as expected		

Eight and four double mutants were constructed in *E. coli* EDL933 and *S.* Typhimurium 14028s, respectively, and tested for synthetic lethality in the indicated growth medium. The nine pairs that did not show synthetic lethality as predicted were classified into one of four classes as follows: *1.* Important biological information was not incorporated into the metabolic model due to inherent limitations of the model; *2.* Missing regulatory information not captured by the model; *3.* At least one of the two genes appears to be singly essential experimentally, but was not singly essential in the model. The singly essential gene is denoted with an asterisk. For the *glyA*/*serA* pair, both genes are singly essential; *4.* Missing biological information, e.g. the possible presence of another unidentified homolog.

**Table 2:** Results of antimicrobial screening of 1,328 compounds against *E. coli* and *S.* Typhimurium.

Target Protein	Number of Compounds Tested	Number of Active Compounds			
		E. coli		S. Typhimurium	
		IC_50_ of 0.1–200 μM	>30% inhibition at 200 μM	IC_50_ of 0.1–200 μM	>30% inhibition at 200 μM
HemN	89	0	1	0	0
SucC	279	0	5	1	3
LpdA	246	0	0	0	0
GlyA	195	0	0	0	0
SerA	154	2	0	2	1
HemF	84	0	0	0	0
Mdh1	203	0	0	0	0
Ppc1	78	0	4	0	1

The table shows the number of compounds tested for each of the target proteins and their respective confirmed activity profile against the two bacteria using repurchased and reweighed samples.

**Table 3:**
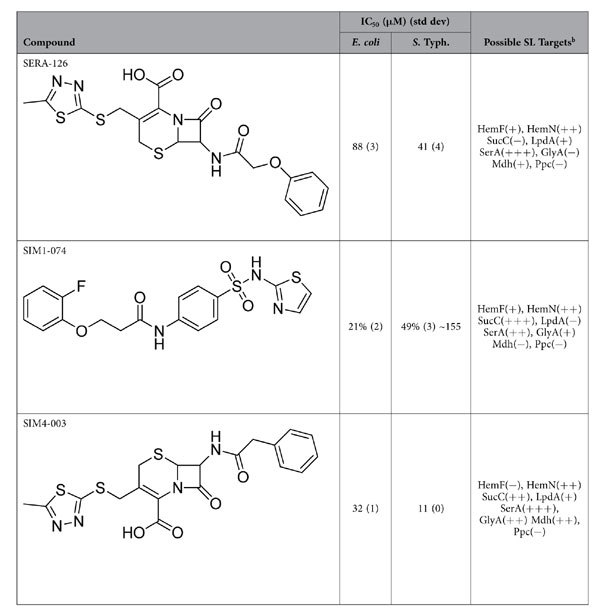
IC_50_ data for hit compounds in bacterial growth inhibition assays, n = 2 with standard deviations to one significant figure in parentheses.

For the weak inhibitor SIM1-074, percent inhibitions at the top dose, 200 μM, are reported. One experiment provided sufficient data to calculate an IC_50_ (155 μM). The possible SL targets for each compound were determined by docking the compounds against each of the eight proteins. Each “+” symbol is one standard deviation above the average of random compounds docked in the binding site. A “-” symbol implies that the compound did not dock into that site or was less than one standard deviation from the random compound average.
