# Supplementary material for: Systems biology-guided identification of synthetic lethal gene pairs and its potential use to discover antibiotic combinations
Source: Sci Rep. 2015 Nov 4;5:16025. doi: 10.1038/srep16025 (PMC4631998; doi:10.1038/srep16025)
Supplement: Supplementary Information 1 [file srep16025-s2.doc]

**SUPPLEMENTARY FILE 1:**

**Detailed materials and methods for virtual and biological screening**

**Systems biology-guided identification of synthetic lethal gene pairs and its potential use to discover antibiotic combinations**

Ramy K. Aziz^1,2^, Jonathan M. Monk^2^, Robert M. Lewis^3^, Suh In Loh^4^, Arti Mishra^4^, Amrita Abhay Nagle^4^, Chitkala Satyanarayana^4^, Saravanakumar Dhakshinamoorthy^4^, Michele Luche^3^, Douglas B. Kitchen^3^, Kathleen A. Andrews^2^, Nicole L. Fong^2^, Howard J. Li^2^, Bernhard O. Palsson^2^, Pep Charusanti^2,5,§^

^1^ Department of Microbiology and Immunology, Faculty of Pharmacy, Cairo University, Cairo, 11562, Egypt

^2^ Department of Bioengineering, University of California, San Diego, La Jolla, California, 92093, USA

^3^ Computer-Aided Drug Discovery, Albany Molecular Research, Inc., Albany, New York, 12203, USA

^4^ Albany Molecular Research Singapore Research Centre, Pte Ltd, Singapore, 117525

^5^ The Novo Nordisk Foundation Center for Biosustainability, Technical University of Denmark, Hørsholm, Denmark

^§^ Corresponding author

**Virtual Screening Procedure**

All proteins were prepared using Schrödinger’s Protein Preparation Wizard. Through the Maestro interface, each structure was pre-processed by assigning bond orders, adding hydrogen atoms, and deleting all water molecules. The Impref minimization was run with a convergence of heavy atoms changes at RMSD 0.30 Å and the force field was OPLS 2005.

Using this prepared structure, the receptor grid was then generated using Schrödinger’s Receptor Grid Generation. A ligand from the crystal structure was chosen to define the receptor. In some cases, the ligand was modeled based on known substrates. Typically, a sample ligand was modeled in order to test the ability of the docking procedure to achieve the correct geometry. The vdW scaling factor was set at 1.0, the partial charge cutoff was set at 0.25, and there were no per-atom scaling factors used. The site for the grid was the centroid of the chosen ligand with a box of 10 Å around it.

This prepared structure was also used as the starting point for Molecular Operating Environment’s (MOE) Structure Preparation and Protonate3D. After any errors in the structures were corrected, Protonate 3D was run at a temperature of 300 K, pH of 7, and a salt of 0.1 using the GB/VI method with a cutoff of 15 Å, a dielectric constant of 1, and solvent constant of 80. The vdW settings were the 800R3 method with a 10 Å cutoff.

High-throughput virtual screening (HTVS) docking was performed on diverse sets of 300,000 to 600,000 ionized compounds from our in house virtual library of 70 million compounds using Schrödinger’s Glide 5.8 using the OPLS 2005 force field. The vdW scaling factor was set at 0.8 and the partial charge cutoff was set at 0.15. Any pose with Coulomb-vdW energy greater than 0.0 kcal/mol was rejected and all other poses were clustered with a RMSD of less than 0.5 Å and a maximum atomic displacement of less than 1.3 Å. Compounds that scored less than 2 standard deviations from the mean Glide score were selected for further work. In some cases the exact region that would be inhibitory was difficult to distinguish and the portion of the site that would best accommodate the substrate was used and provided the best opportunity for interactions between potential inhibitors and the binding site. In other cases two sites were used for docking. Any compound that scored well against a protein crystal structure was assigned that possible mechanism of action.

Compounds that scored well from the HTVS run were used as the probe compounds for chemical similarity searching of the entire 70M compound library. Atom pair similarity^1^ calculations were used to determine chemically similar structures with a similarity cutoff of 70-80%. Compounds that contained the maximum HierS scaffolds^2^ of any compound on the HTVS list were also selected. The similarity cutoff was adjusted to yield approximately 50 thousand compounds that were then docked into the site using a higher precision (SP) and lower throughput method. The combined results of the similarity search, scaffold search, and the original compounds from HTVS were combined and filtered using the following criteria: molecular weight <= 600, Oprea number of rotatable bonds <= 10, logP <=4, number of heavy atoms >= 10, Lipinski acceptors <= 10, Lipinski donors <= 10, Lipinski acceptors plus Lipinski donors >=2, TPSA <=200, and no reactive or pains filters set ^3^ SP docking was performed using Schrödinger’s Glide 5.8 using a force field of OPLS 2005. Rings were sampled with an energy window of 2.5 kcal/mol. 1000 maximum initial poses were kept, with the scoring window of 100 and up to 400 kept for minimization. The poses were minimized with a distance-dependent dielectric constant of 2 for a maximum of 100 steps. The ligand vdW scaling factor was set at 0.8 and the partial charge cutoff was set at 0.15. Postdock was performed on 5 poses per ligand and any pose with Coulomb-vdW energy greater than 0.0 kcal/mol was rejected and all other poses were clustered with a RMSD of less than 0.5 Å and a maximum atomic displacement of less than 1.3 Å. The resulting poses were also rescored using the MOE prepared structure and the GBVI-WSA scoring method.

Compounds that scored more than 1 standard deviation lower than the mean in both the Glide score and MOE score and the 20 best scoring compounds from each method were then examined manually to remove any undesirable compounds that were not removed by filtering. These compounds were then sourced for purchasing.

Pdb files for each site

*glyA*: PDB codes 1eqb and 1eqn were used to develop models with a transition state structure for the ligand.

195 compounds were purchased. None had measurable IC_50_ data.

*hemF*: A homology model was created using PDB code 1vju as the template and the *S.* Typhimurium sequence. Half of the natural substrate, coproporphyrinogen III, was used as the ligand.

84 compounds were purchased. Two compounds had measurable IC_50_ values:

HEMF-053: 170.1 µM against *E. coli*, 157.2 µM against *S.* Typhimurium.

HEMF-052: 163.1 µM against *E. coli*, 177.2 µM against *S.* Typhimurium

*hemN:* PDB code 1olt was used to develop a model using SAM as the ligand.

A total of 89 compounds were purchased for testing. Two compounds had measurable IC_50_ values:

HEMN-007: 26.8 µM against *E. coli*, 27.5 µM against *S.* Typhimurium.

HEMN-009: 25.6 µM against *E. coli*, 24.6 µM against *S.* Typhimurium.

*lpdA:* A homology model was created using PDB code 1ojt as the template and the *S.* Typhimurium sequence. PDB ligand 3ii was used as the ligand.

A total of 246 compounds were purchased for testing. None had measurable IC_50_ data.

*mdh:* PDB codes 2cmd and 3hhp were used to develop models. With 3hhp, the NAD and CIT from 1emd were included in the model and CIT was used as the ligand. For 2cmd, the NAD from 1ib6 was included. This clashed with 2cmd’s CIT, so the adenosine portion was used in the model, with CIT being used as the ligand.

A total of 203 compounds were purchased for testing. None had measurable IC_50_ data.

*ppc:* PDB code 1jqn was used to develop two models. One model used DCO as the ligand and the Mn was removed for the main site. In the second model, the ASP served as the ligand for the allosteric site.

A total of 78 compounds were purchased for testing. Five had measurable IC_50_ values.

PPC1-007: 189 μM in *E. coli*, 158 μM in *S.* Typhimurium

PPC1-008: 152 μM in *E. coli*, 168 μM in *S.* Typhimurium

PPC1-009: 188 μM in *E. coli*, 130 μM in *S.* Typhimurium

PPC1-018: 119 μM in *E. coli*

PPC1-019: 195 μM in *E. coli*

*serA:* PDB code 1yba was used to develop models. First we used AKG as the ligand with NAD removed, second with both AKG and NAD used as the ligand, and third with AKG as the ligand and NAD in the model.

A total of 154 compounds were purchased for testing. Two had measurable IC_50_ values.

SERA-126: 125.89 µM in *E. coli*, 100.68 µM in *S.* Typhimurium.

SIM4-003: 32.24 µM in *E. coli*, 11.31 µM in *S.* Typhimurium.

*sucC*: PDB code 1jkj was used to develop a model using the Suc-CoA-based ligand below (**b**).

A total of 279 compounds were purchased for testing. None had measurable IC_50_ values.

A total of 67 hits were identified from the cell culture assays with > 20% inhibition in either cellular assay. Atom-pair similarity calculations were performed using the 67 compounds as probe structures in four rounds of searches (sim1-sim4) to identify additional structures that might bind to the target proteins. After docking, 289 additional compounds were purchased. Table S-1 summarizes the distribution of predicted targets.

Table S-1. Summary of number of compounds purchased by stage and by protein target.

|  | Search Stage | |  |
| --- | --- | --- | --- |
|  | Round 1 | Sim1-4 | Total |
| *hemN* | 51 | 38 | 89 |
| *sucC* | 147 | 132 | 279 |
| *lpdA* | 201 | 45 | 246 |
| *glyA* | 195 | 0 | 195 |
| *serA* | 153 | 1 | 154 |
| *hemF* | 70 | 14 | 84 |
| *mdh1* | 144 | 59 | 203 |
| *ppc1* | 78 | 0 | 78 |

| *Similarity*  *Round* | No. of  Compounds |
| --- | --- |
| *sim1* | 108 |
| *sim2* | 99 |
| *sim3* | 59 |
| *sim4* | 23 |

**Software**

1. Schrödinger Suite 2011 Schrödinger Suite; Glide version 5.8, Schrödinger, LLC, New York, NY, 2012
2. Molecular Operating Environment (MOE), 2012.10; Chemical Computing Group Inc., 1010 Sherbooke St. West, Suite #910, Montreal, QC, Canada, H3A 2R7, 2012.

**Biological Screening Procedure**

Bacterial strains and other materials: Glycerol [G7893], Sodium Chloride [S271-1], DMSO [472301], Amikacin [A3650], Ceftriaxone [C5793], Chloramphenicol [C1863], Levofloxacin [28266], Azithromycin [75199]; and Tetracycline [T3383] were purchased from Sigma-Aldrich, Inc. (St. Louis, MO). Cation-adjusted Mueller-Hinton (MH2) broth [212322], Cation-adjusted Mueller-Hinton (MH2) agar [211438] were purchased from BD Biosciences (San Jose, CA). Corning polystyrene clear 384-well sterile plates [07-201-157], Corning polypropylene 384-well storage plates [07-200-747], Corning polystyrene clear 96-well sterile plates [07-200-656], and Corning polypropylene 96-well storage plates [07-200-695] were purchased from Thermo Fisher Scientific Inc. (Waltham, MA). Hummingbird (Cartesian), Wellmate micro plate dispenser with stacker (Matrix Technologies) and Biomek Fx (Beckman Coulter) were used for compound handling and dispensing the bacterial strains. Analyst GT plate reader (Molecular Devices) was used for reading the assay plates for absorbance.

Propagation of bacterial strains, CFU enumeration and glycerol stock preparation: Mueller Hinton Broth (MHB) was prepared by dissolving 22g of MHB in 1000mL of double distilled water and boiled for 1 minute. The solution was autoclaved at 121°C, 15 PSI for 20 minutes and stored at room temperature. Mueller Hinton Agar (MHA) was prepared by dissolving 19g of MHA in 500mL of double distilled water and autoclaved at 121°C, 15 PSI for 20 minutes. The solution was allowed to cool to 55°C in a water bath and 20mL of the MHA was poured into each petri dish. The agar plates were stored at 4°C. All bacterial cultures were handled according to NIH Biosafety Level 2 guidelines, with all culture manipulations restricted to Class II biological safety cabinets. The bacterial strains were cultured on MHA and MHB at 37° C for 18 hours. Fresh glycerol stocks (10%) were made for each strain and stored at -80° C. Colony-forming units (CFUs) were determined using standard protocols for frozen glycerol stocks and assay inocula of all strains to assess initial viability and to ensure consistent assay performance. Briefly, for each strain, the cryovial was thawed and serially diluted (1:9) in saline in two parallel columns in a sterile 96-well polystyrene plate. Starting with the highest dilution, three 20 µL spots were dispensed from each well of the dilution plate to the corresponding quadrant on one of the agar plates. After spots were completely dry, the plates were incubated overnight at 37°C for up to 20h. Average numbers of colonies per spot in each countable quadrant were determined by using all six spots (3 spots over 2 agar plates) and CFU per mL of the cryovial was determined using the following calculation. (Average number of colonies * 1000/20) * (10^Quadrant^).

Preparation of compound master plate for primary screening and to determine the IC_50_ and % inhibition: 12.8 mg/mL solution of the standard antibiotics amikacin, ceftriaxone, chloramphenicol, levofloxacin, tetracycline and azithromycin were prepared in 100% DMSO and stored at -20°C. A 20mM stock of each of the test compounds was prepared in 100% DMSO. A four point serial dilution (1:8) of the standard antibiotics and test compounds were prepared in a 96-well plate. Transfer of the compounds and controls (DMSO) to the 384 well master plates was performed using the Biomek Fx. The compounds were further serially diluted (1:2) in 100% DMSO using the Biomek Fx. The concentration range achieved for standard antibiotics was 12.8 mg/mL to 0.003 mg/mL. The concentration range achieved for the test compounds was 200 µM to 1.56 µM. The plate was centrifuged at 1000 rpm for 1 minute and stored at -20°C until addition into the assay plate.

Primary screening / IC_50_ determination: Compounds were transferred from the compound master plate to the assay plate using the Humming bird. The required top test concentration determined the number of transfers performed. The glycerol stocks of the microbial strains were thawed in the biological safety cabinet and added to the MHB (as per CLSI guidelines, final concentration of 5x10^5^ CFU per mL was used). 50 μL of prepared inoculum was added to the assay plates (column 3-24) within 10 minutes of preparation. Columns 1-2 were used as the negative controls where the growth media (without inoculum) was added. Plates were incubated at 37°C for 18 hours. After 18 hours, the plates were incubated at room temperature for 30 minutes before determining the absorbance at 600nm using Analyst GT. Data analysis was done using the Excel Fit software form IDBS.

Combination studies: Combination study was performed using 3 compounds (SERA-126, SIM1-074 and SIM4-003) selected from the primary screening. Two concentrations of SIM1-074 were tested against two concentrations of SERA-126 and SIM4-003. Concentrations that caused 25 and 50 percent growth inhibition of *E. coli* and/or *S.* Typhimurium were chosen for the combination study. The compound master and assay plates were prepared as described in the previous sections.

**BIBLIOGRAPHY**

1 Carhart, R. E., Smith, D. H. & Venkataraghavan, R. Atom Pairs as Molecular-Features in Structure Activity Studies - Definition and Applications. *J Chem Inf Comp Sci* **25**, 64-73, doi:Doi 10.1021/Ci00046a002 (1985).

2 Wilkens, S. J., Janes, J. & Su, A. I. HierS: Hierarchical scaffold clustering using topological chemical graphs. *J Med Chem* **48**, 3182-3193, doi:Doi 10.1021/Jm049032d (2005).

3 Baell, J. B. & Holloway, G. A. New substructure filters for removal of pan assay interference compounds (PAINS) from screening libraries and for their exclusion in bioassays. *J Med Chem* **53**, 2719-2740, doi:10.1021/jm901137j (2010).
